# Clinical Characteristics of Cognitive Subgroups of Obsessive Compulsive Disorder

**DOI:** 10.1002/brb3.70375

**Published:** 2025-03-13

**Authors:** Emre Mısır, Raşit Tükel, Berna Binnur Akdede, Emre Bora

**Affiliations:** ^1^ Department of Psychiatry, Faculty of Medicine Baskent University Ankara Turkey; ^2^ Department of Interdiciplinary Neuroscience, Graduate School of Health Sciences Ankara University Ankara Turkey; ^3^ Department of Psychiatry, Faculty of Medicine İstanbul University İstanbul Turkey; ^4^ Department of Psychiatry, Faculty of Medicine Dokuz Eylül University İzmir Turkey; ^5^ Department of Neurosciences, Health Sciences Institute Dokuz Eylül University İzmir Turkey

**Keywords:** cognitive clusters, heterogeneity, neurocognition, obsessive compulsive disorder

## Abstract

**Introduction:**

Obsessive‐compulsive disorder (OCD) is a clinically heterogeneous disorder. The results of symptom‐based classification studies are inconsistent in resolving this heterogeneity. The aim of this study was to investigate clinical differences between clusters created according to neurocognitive performance.

**Methods:**

This study combined data sets from three previously published studies. A total of 135 outpatients diagnosed with OCD, and 106 healthy controls (HCs) were evaluated using the 17‐Item Hamilton Depression Rating Scale (HDRS‐17) and a comprehensive neuropsychological battery. Patients were also administered the Yale‐Brown Obsessive Compulsive Scale (Y‐BOCS).

**Results:**

Two neurocognitive subgroups were identified by *k*‐means cluster analysis: globally impaired (GI, *n* = 42) and cognitively intact (CI, *n* = 93). The GI subgroup performed worse than the HC and CI groups on all neurocognitive tests. There was no difference between the CI group and HC in any cognitive domains. Compulsive symptom severity [*t*(133) = −2.45, *p* = 0.015], Y‐BOCS total score [*t*(133) = −2.09, *p* = 0.038], and age of onset were higher in the GI group than in the CI group [*t*(132) = −4.24, *p* < 0.001]. Years of education were higher in the CI and HC groups than in the GI group [*F*(238) = 35.27, *p* < 0.001]. There was no difference in symptom profile between the CI and GI groups.

**Conclusion:**

The identified cognitive clusters may indicate subtypes with different neurobiological bases. A better dissection of the cognitive structure of OCD could potentially facilitate genetic and neuroimaging studies.

## Graphical abstract

Obsessive‐compulsive disorder has been classified into two subgroups based on neurocognitive performance: globally impaired (GI) and cognitively intact (CI). In the GI subgroup, total symptom severity, compulsive symptom severity, and age of onset of illness were higher, while years of education were lower. HCs, healthy controls; OCD, obsessive‐compulsive disorder; ProcSpeed, processing speed; VerMem, verbal memory; WM, working memory, Executive, executive function, Fluency, verbal fluency

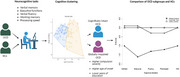

## 1 Introduction

Obsessive‐compulsive disorder (OCD) is a psychiatric disorder characterized by obsessions and compulsions with a lifetime prevalence of 1%–3% in the population (Karno et al. 1988; Mahjani et al. 2021). Studies with large sample sizes have demonstrated that obsessions and compulsions can be categorized into three to six factors (Bloch et al. 2008; Mataix‐Cols et al. 2005). Furthermore, several studies suggest that different symptom profiles vary in their neurobiological basis and clinical features such as age of onset (AoO), insight and prognosis, but the results are contradictory (Alarcon et al. 1993; Mataix‐Cols et al. 1999; Lee et al. 2009; de Wit et al. 2014; Okada et al. 2015; Fouche et al. 2017; Hirose et al. 2017; Yagi et al. 2017; Bragdon et al. 2018). At the same time, although the increased family risk of OCD has been shown to be largely (47%) attributable to genetic factors without a significant influence of shared environment, the results of genome‐wide association studies have not been replicated (Mataix‐Cols et al. 2013; Stewart et al. 2013; Arnold and Brentani 2018). The challenges in explaining the etiopathogenesis of the disease may stem from its clinical heterogeneity (Abramovitch et al. 2019; Stein et al. 2019). Because multiple symptoms co‐occur and fluctuate over time, categorization at the symptom level may lead to confounding results. Instead, classifications based on neuroimaging and neurocognitive assessments may be more effective in creating more homogeneous groups (Insel et al. 2010).

Numerous neuroimaging studies have shown that abnormalities in the cortico‐striato‐thalamo‐cortical (CSTC) circuit are associated with OCD (Piras et al. 2013; Barahona‐Corrêa et al. 2015; Attwells et al. 2017; Hazari et al. 2019; Goodman et al. 2021). It is thought that gray and white matter alterations in the CSTC are related to a decrease in top‐down inhibition, leading to excessive activity associated with self‐referential processes and habit formation in motor and cognitive circuits (Stein et al. 2019; Amaya and Smith 2018). Disruption of neural circuits also likely contributes to cognitive, emotional, and behavioral changes (Rasgon et al. 2017). Therefore, in addition to being a consequence of neural changes, impaired neurocognitive functions such as cognitive inflexibility, reduced inhibition, and set‐shifting may serve as intermediate markers for understanding the underlying causes of OCD symptoms (Bora 2020).

OCD is characterized by a widespread neurocognitive impairment profile (Barahona‐Corrêa et al. 2015; Snyder et al. 2015). Reduced cognitive flexibility and the inability to inhibit repetitive behaviors and thoughts play a key role in the formation of obsessions and compulsions (Gruner and Pittenger 2017). The neurocognitive profile of OCD reflects abnormalities in the frontostriatal circuitry, involving a dominance of the direct pathway in the basal ganglia motor circuit (Piras et al. 2013). Animal models also indicate that striatal dysfunction is associated with increased repetitive behaviors, heightened grooming behavior, and impaired inhibition of learned behaviors (Lu et al. 2021). Numerous studies have demonstrated that OCD is related to worse performance in set‐shifting, response inhibition, working memory, verbal memory, visuospatial abilities, and motor speed (Abramovitch et al. 2019; Snyder et al. 2015; Abramovitch et al. 2013; Shin et al. 2014). According to a meta‐analysis by Shin et al. (2014), impairments in planning, visual memory‐immediate recall, and visuospatial organization were observed with medium to large effect sizes, whereas deficits in verbal memory, verbal fluency, processing speed, and set‐shifting were found to have small to medium effect sizes (Shin et al. 2014). Another meta‐analysis reported medium effect sizes in executive functions, including planning, set‐shifting, and response inhibition (Abramovitch et al. 2013). Small effect sizes were found for visuospatial abilities, working memory, and verbal memory, but a large effect size was observed for nonverbal memory. In a recent meta‐analysis of studies comparing OCD patients, their relatives, and healthy controls (HCs), patients were found to perform worse than healthy individuals in all neurocognitive domains (Bora 2020).

Although studies suggest impairment in multiple cognitive domains in OCD, patients with different levels of cognitive impairment may differ clinically. Data‐driven cluster analysis according to neurocognitive abilities may help to detect subtle differences among patients with OCD and identify different phenotypes. There are many studies showing that clusters with cognitively distinct characteristics also differ clinically in patients with schizophrenia, bipolar disorder, and major depressive disorder (MDD) (Burdick et al. 2014; Lewandowski et al. 2014; Bora et al. 2016; Vicent‐Gil et al. 2021; Bora et al. 2023; Guo et al. 2023). However, to the best of our knowledge, there is only one study that has compared cognitive subgroups of a clinically heterogeneous disorder such as OCD (Malcolm et al. 2021). In the study, the mixed patient group consisting of OCD and body dysmorphic disorder diagnoses was divided into two clusters: intact cognition and severely impaired cognition. The study found no clinically significant difference between the cognitive clusters. On the other hand, there is a need for a study with a larger sample size consisting only of patients with OCD.

The main objective of this study is to examine clinical differences between cognitive subgroups created using a data‐driven approach. Our main hypothesis is that higher symptom severity is associated with severe cognitive impairment.

## 2 Experimental Procedures

### 2.1 Study Selection

Data sets from three previously published studies that met specific criteria were used in this study. Firstly, the PubMed, PubMed Central, and Scopus databases were initially searched by E.M. using the keyword combination: *“obsessive‐compulsive disorder”* AND (cogn* OR neuropsych*) to identify eligible studies (published between January 1, 2003, and August 10, 2023). Google Scholar and cross‐references were also endorsed to identify additional studies. The inclusion criteria for studies were: (i) Turkish adult patients with OCD and HCs were investigated, (ii) utilized a comprehensive neurocognitive test battery including verbal memory, processing speed, executive functions, working memory, and verbal fluency domains. Seven studies were identified that met these criteria and overlapped in the cognitive domains assessed (Kıvırcık Akdede et al. 2005; Tükel et al. 2012; Tükel et al. 2012; Kartal 2015; Mısır et al. 2018; Ozcan et al. 2016). Letters of invitation to share data were sent to the authors, and studies with responding authors were included (Kıvırcık Akdede et al. 2005; Tükel et al. 2012; Mısır et al. 2018).

This study was approved by the Baskent University Institutional Review Board and Ethics Committee (Project no and date: KA24/310/18.09.2024).

### 2.2 Participants

In this study, data from three different studies conducted at two centers were combined. Studies 1 and 2 were conducted at Dokuz Eylül University Department of Psychiatry (Center 1), and no overlap was found between their samples (Kıvırcık Akdede et al. 2005; Mısır et al. 2018). Study 3 was conducted at the Istanbul University Department of Psychiatry (Center 2) (Tükel et al. 2012). In addition, data from seven additional patients available to the authors but not initially included in Study 3 were also included in this study. A total of 135 adult patients with OCD and 106 HCs were enrolled. All patients had a diagnosis of OCD based on the Structured Clinical Interview for DSM‐IV/Clinical Version. In all studies, the exclusion criteria for patients were: (i) the presence of a neurological disorder or a medical condition that might impair cognitive performance, (ii) comorbid MDD, bipolar disorder, and personal history of schizophrenia spectrum disorders (excluding schizotypal personality disorder), (iii) alcohol and/or drug abuse or dependence, and (iv) use of benzodiazepines. Study 3 included drug‐free patients for at least 6 weeks before the recruitment. In all studies, HCs with no any lifetime or current psychiatric disorder, no family history of overt psychotic disorder, bipolar disorder, or OCD were enrolled.

### 2.3 Clinical Assessments

The Yale‐Brown Obsessive Compulsive Scale (Y‐BOCS) was administered to assess the severity of illness, and the Y‐BOCS symptom checklist was used to assess symptom types in patients (Goodman et al. 1989). Individuals with one or more primary obsessions related to aggression, religion, or sexuality were included in the autogenous obsessions group, while individuals with one or more primary obsessions related to contamination, suspicion, symmetry, somatic concerns, or hoarding were included in the reactive obsessions group. The Hamilton Depression Rating Scale (HDRS‐17) was used to assess the severity of depressive symptoms in all participants (Hamilton 1960).

### 2.4 Neurocognitive Assessments

Neurocognitive test results that were shared by at least two of the three studies were included in the analyses. Study 3 used the California Verbal Learning Test (CVLT), but others have used the Rey Verbal Learning Test (RAVLT) to assess verbal learning (Delis et al. 2000; Lezak 2004). RAVLT scores were converted to CVLT scores to ensure standardization across all participants (Kennedy et al. 2023). CVLT Trial 1, learning (Trials 1–5), and delayed recall (Trial 7) scores were used to assess verbal memory. Executive functions were measured using the Wisconsin Card Sorting Test (WCST) and the Trail Making Test‐B (TMT‐B) (Spreen and Strauss 1998). Outcome variables included the number of completed categories and perseverative errors from the WCST and the total time taken to complete the TMT‐B. The time taken to complete the TMT‐A was used to measure processing speed (Spreen and Strauss 1998; Reitan 1958). The Controlled Oral Word Association Test (COWAT; letters K, A, S) was used to measure verbal fluency (Spreen and Strauss 1998). To assess working memory, raw scores of the digit span task (DST, forward and backward) and the total score of the ACTT were the outcome variables (Lezak 2004). The Auditory Consonant Trigram Test (ACTT) and the DST were not measured in one study (Tükel et al. 2012). Therefore, data from two other studies were used to assess the working memory domain (56 patients and 52 HCs). Five cognitive domain composite scores (z‐scores) of patients were also calculated based on the performance of HCs: (1) verbal memory, (2) executive functions, (3) verbal fluency, (4) working memory, and (5) processing speed.

### 2.5 Data Analysis

*k*‐means cluster analysis was used to identify cognitive clusters. The variables used in this analysis were verbal memory, executive functions, verbal fluency, and processing speed. The number of clusters was determined by examining gap statistics. Comparisons between groups according to sociodemographic, clinical, and neurocognitive variables were made using one‐way analysis of variance (ANOVA), independent samples *t* test, and chi‐squared tests. For post‐hoc comparisons, the Tukey test was used for ANOVA, and the Bonferroni correction was used for chi‐squared test. The effects of age and gender on the difference in neurocognitive variables between cognitive subgroups were controlled with ANCOVA. Effect sizes were calculated based on partial eta squared for ANOVA, and Cohen's d for *t* test. Statistical analyses were performed using JAMOVI and SPSS version 22 (SPSS, Chicago, IL, USA).

## 3 Results

### 3.1 Demographic and Clinical Characteristics

The study included 135 patients [age = 29.53 (9.53), 99 female, 65.9%] and 106 HCs [age = 29.91 (9.46), 56 female, 52.8%]. There were no differences in age or marital status between the patient and HCs. Patients were significantly more likely to be female and unemployed. In addition, patients had a lower level of education compared to the HC group (see Table S1).

Cluster analysis indicated that a two‐cluster solution was the most appropriate (Figures S1 and S2). The first cluster (globally impaired [GI] cognition) included 42 patients (31%), whereas the second cluster (cognitively intact [CI] cognition) included 93 patients (69%). The GI group was significantly older than both the CI group (*p* = 0.001) and the HC group (*p* = 0.045). There was no gender difference between the CI and HC groups, but the GI group had a significantly higher proportion of females (corrected *p* = 0.015). Although initial analysis indicated significant differences in marital status between the cognitive performance groups, after Bonferroni correction for the chi‐square test, the groups were found to have similar marital status distributions. The CI (*p* < 0.001) and HC groups (*p* < 0.001) had significantly more years of education than the GI group. There were no significant differences in employment status between the groups.

Patients in the impaired group had significantly higher AoO, Y‐BOCS compulsion, and Y‐BOCS total scores (see Table 1). Thirty‐three patients were on antidepressant monotherapy (9 of GI and 24 of CI) and eight patients were on antidepressant and antipsychotic combination (5 of GI and 3 of CI). Rates of medication use and severity of depressive symptoms were similar between the groups.

**TABLE 1:** Demographic and clinical characteristics of cognitive groups.

	Globally impaired (*n* = 42)	Cognitively intact (*n* = 93)	HCs (*n* = 106)	Statistics	Post‐hoc
Age	34.79 (11.48)	27.15 (7.44)	29.91 (9.46)	*F*(238) = 8.63 *p* < 0.001	GI > CI = HC
Gender (F/M)	34/8	55/38	56/50	*χ* ^2^ (2) = 9.99 *p* = 0.007^**^	
Education	7.76 (3.27)	12.63 (3.34)	12.51 (4.44)	*F*(238) = 35.27 *p *< 0.001	CI = HC > GI
Married/no married^a^	21/21	29/64	50/56	*χ* ^2^ (2) = 6.73 *p* = 0.035	—
Employed/unemployed	16/26	30/63	51/55	*χ* ^2^ (2) = 5.28 *p* = 0.07	—
AoO	25.96 (8.82)	20.46 (5.71)		*t*(132) = −4.24 *p *< 0.001	
Disease duration	8.82 (8.46)	6.59 (6.86)		*t*(132) = −1.62 *p* = 0.11	
Medication (Yes/No)	14/28	27/64		*χ* ^2^ (1) = 0.09 *p* = 0.76	
Y‐BOCS total	25.93 (6.35)	23.24 (7.15)		*t*(133) = −2.09 *p* = 0.038	
Y‐BOCS‐O	12.95 (3.99)	12.06 (3.59)		*t*(133) = −1.35 *p* = 0.18	
Y‐BOCS‐C	13.21 (3.92)	11.27 (4.41)		*t*(133) = −2.45 *p* = 0.015	
HDRS‐17	6.64 (3.95)	6.22 (4.09)	1.6 (2)	*F*(238) = 60.49 *p*<0.001	CI = GI > HC

Abbreviations: F/M, Feamale/Male ratio, AoO, age of onset; HDRS‐17, 17‐item Hamilton Depression Rating Scale; Y‐BOCS, Yale‐Brown Obsession and Compulsion Scale; Y‐BOCS‐C, Y‐BOCS compulsion subscale; Y‐BOCS‐O, Y‐BOCS obsession subscale.

^a^ No significant difference after the Bonferroni correction in the post‐hoc comparison.

There was no significant difference between the GI and CI groups in the types of obsessions and compulsions (see Table 2).

**TABLE 2:** Obsessive and compulsive symptoms in patients.

Obsessions	Globally impaired	Cognitively intact	*χ* ^2^	*p*
Aggressive (Yes/No)	4/38	9/84	0	1
Contamination (Yes/No)	36/6	70/23	1.31	0.25
Sexual (Yes/No)	6/36	16/77	0.03	0.8
Hoarding/saving (Yes/No)	4/42	10/83	0	1
Religious (Yes/No)	10/32	35/58	1.91	0.17
Symmetry/exactness (Yes/No)	18/24	31/62	0.76	0.34
Somatic (Yes/No)	6/36	12/81	0	1
Compulsions				
Cleaning/washing (Yes/No)	35/7	70/23	0.67	0.41
Checking (Yes/No)	23/19	53/40	0.003	0.96
Repeating rituals (Yes/No)	14/28	21/72	1.23	0.27
Counting (Yes/No)	7/35	73/20	0.67	0.41
Ordering/arranging (Yes/No)	14/28	29/64	0.002	0.96
Hoarding/collecting (Yes/No)	5/37	10/83	0	1
Autogenous obsessions (Yes/No)	7/35	11/82	0	1
Reactive obsessions (Yes/No)	27/15	45/48	0	1

### 3.2 Neurocognitive Characteristics

**FIGURE 1:**
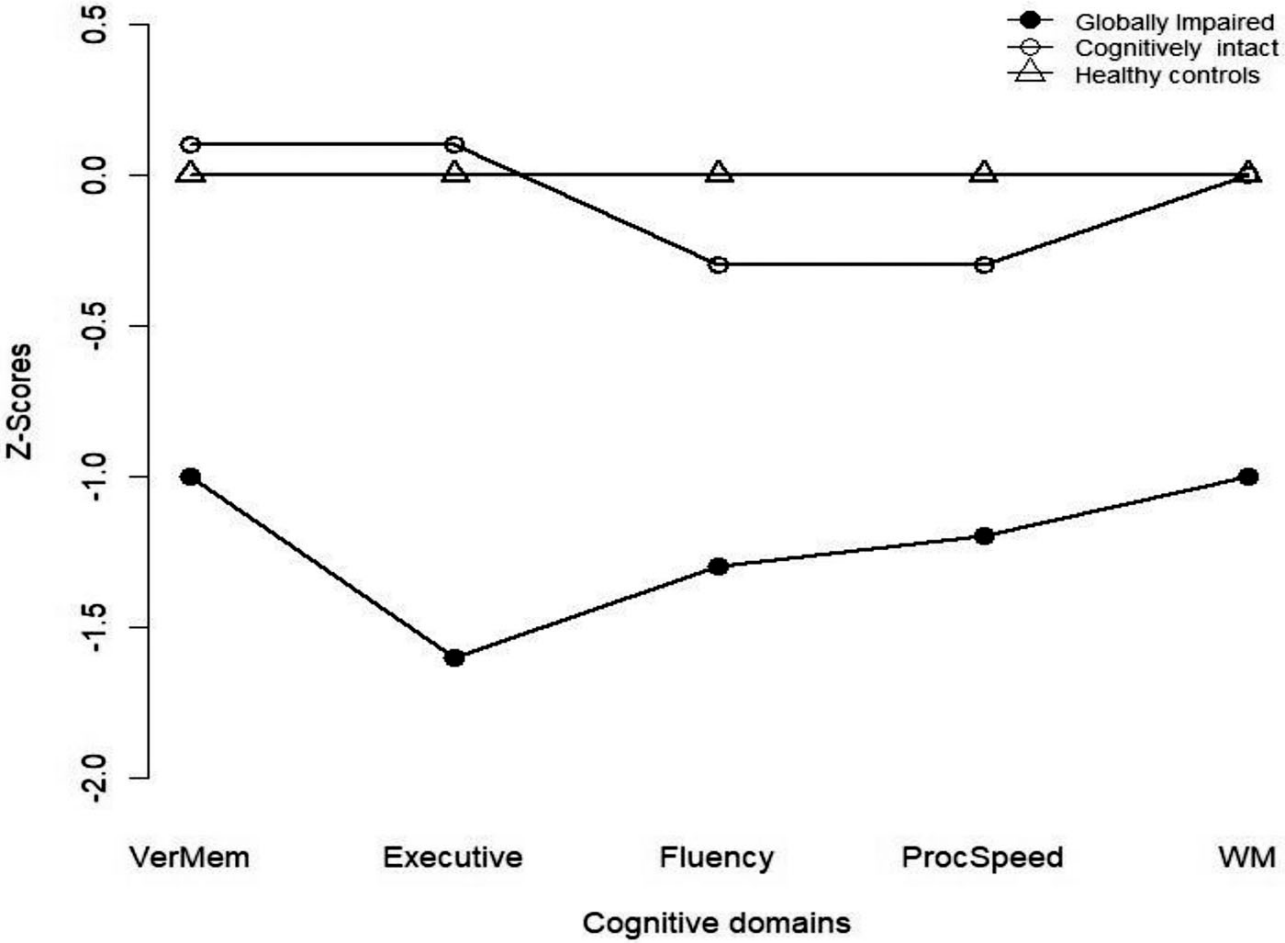
z‐scores of cognitive domains of globally impaired and cognitively intact subgroups according to healthy controls. ProcSpeed, processing speed; VerMem, verbal memory; WM, working memory.

There was no difference between patients and HCs in CVLT delayed recall, WCST category number, ACC total score, and Digit Span Forward Test scores (see Table S2). Patients were worse on other subtests and in all cognitive domains. In comparisons between cognitive clusters, there were no significant differences between the CI and HC groups on any neurocognitive test except for the number of categories completed on the WCST. The GI group performed worse than the other two groups on all neurocognitive tests and cognitive composite scores (see Table 3 and Figure 1). The number of WCST categories in the CI group was significantly higher than in the HC group (*p* = 0.03). Effect sizes in between‐group comparisons were small for the Digit Span Forward and Digit Span Backward tests; medium for the TMT‐A and ACTT; and large for other tests assessing executive functions, verbal memory, and verbal fluency. In five cognitive domains, there was no significant difference in performance between the CI and HC groups, while the GI group had the lowest scores (see Table 3). The results remained unchanged after controlling for age and gender, except for verbal fluency (*F*_corrected_ in Table 2). After controlling for age and gender, HCs had the highest scores in verbal fluency, followed by the CI group, and the GI group had the lowest scores (HCs > CI > GI). Regarding the cognitive domains, the effect size was large for verbal memory, executive functions, verbal fluency, and processing speed, while it was medium for working memory.

**TABLE 3:** Neurocognitive performances in cognitive subgroups and healthy controls.

	Globally impaired (*n* = 42)	Cognitively intact (*n* = 93)	HC (*n* = 106)	*F*	*F*_corr_	*p*	*ηp* ^2^	Post‐hoc
Verbal memory	−1.0 (0.7)	0.1 (0.6)	0 (1.0)	28.5	21.8	< 0.001	0.16	CI = HC > GI
CVLT Trial 1	5.3 (1.4)	7.5 (1.9)	7.5 (2.4)	19.9		< 0.001	0.14	CI = HC > GI
CVLT Learn	44.6 (9.6)	55.5 (8.5)	56.1 (11.3)	21.7		< 0.001	0.15	CI = HC > GI
CVLT Del	9.4 (2.9)	12.8 (2.0)	12.2 (2.9)	25.4		< 0.001	0.18	CI = HC > GI
Executive functions	−1.6 (0.9)	0.1 (0.5)	0 (1)	83.1	67.4	< 0.001	0.36	CI = HC > GI
WCST Per	31.2 (13.3)	12.9 (6.8)	15.1 (10.5)	52.4		< 0.001^**^	0.31	CI = HC > GI
WCST Cat	2.3 (1.6)	5.4 (1.2)	4.8 (1.6)	61.6		< 0.001	0.34	CI> HC > GI
TMT‐B Dur	166.7 (89.5)	84.8 (42.7)	79.8 (48.9)	39.7		< 0.001^**^	0.25	CI = HC > GI
Verbal fluency	−1.3 (0.5)	−0.3 (0.9)	0 (1.0)	30.9	22.6	< 0.001	0.16	CI = HC > GI
COWAT	23.4 (8.0)	38.6 (13.7)	43.0 (15.4)	30.9		< 0.001	0.21	CI = HC > GI
Processing speed	−1.2 (1.3)	−0.3 (0.9)	0 (1.0)	20.2	13.4	0.001	0.10	CI = HC > GI
TMT‐A Dur	51.7 (21.2)	36.4 (14.8)	32.3 (16.4)	20.2		< 0.001^**^	0.14	CI = HC > GI
Working memory	−1.0 (0.6)	0.0 (0.6)	0 (1.0)	11.6	6.1	0.003	0.11	CI = HC > GI
ACTT total	39.9 (8.1)	51.7 (6.7)	49.9 (8.3)	14.5		< 0.001	0.11	CI = HC > GI
DS‐F	4.7 (1.4)	6.6 (2.1)	6.9 (2.6)	5.9		0.004	0.05	CI = HC > GI
DS‐B	4.5 (1.7)	6.6 (2.2)	7.2 (3.0)	7.0		0.001	0.06	CI = HC > GI

*Note*: *F*_corr_ = ANCOVA analysis corrected for sex and age.

Abbreviations: ACTT, Auditory Consonant Trigram Test; CI, cognitively intact; COWAT, Controlled Oral Word Association Test; CVLT, California Verbal Learning Test; CVLT Del, CVLT Delayed recall (Trial 7); CVLT Learn, CVLT Learning (Trials 1–5); DS‐B, DS backward test; DS‐F, Digit Span Forward Test; GI, globally impaired; HC, healthy controls; TMT‐B Dur, Trail Making Test duration to complete; WCST, Wisconsin Card Sorting Test; WCST Cat, WCST completed category; WCST Per, WCST perseverative errors.

There was no significant difference between males and females in any of the cognitive composite scores in either the CI or GI groups. Significant correlations were found between the AoO and processing speed in both the GI group (*r* = −0.31, *p* = 0.049) and the CI group (*r* = −0.22, *p* = 0.034). There were significant positive correlations between years of education and cognitive domain scores (*r* = 0.45–0.68, all *p* < 0.001). Therefore, the covariate effect of years of education could not be assessed.

## 4 Discussion

The current study investigated the clinical characteristics of cognitive subgroups within OCD. The results of the study point to cognitive heterogeneity in OCD. Cluster analysis revealed two cognitive profiles: GI and CI. Total obsessive‐compulsive symptom severity and compulsive symptom severity were higher in the GI group. However, the symptom frequencies were similar between the cognitive subgroups. The GI group had lower years of education, was more likely to be female, and had a higher AoO.

The primary finding of this study indicates cognitive heterogeneity in OCD. The group identified as CI by cluster analysis was found to be similar to HCs on both neurocognitive tests and cognitive domains. In addition, the number of categories completed on the WCST was higher in the CI subgroup. The higher number of categories completed, despite no difference in perseverative errors between the two groups, suggests that the CI subgroup has the ability to recognize perseverative errors more quickly and shift to the correct strategy. Increased self‐monitoring and error‐related negativity (ERN) associated with error detection have been proposed as core endophenotypes for OCD (Barahona‐Corrêa et al. 2015; Bellato et al. 2021). The ERN is interpreted as a signal that triggers behavioral adjustments to enhance performance and prevent future errors (Riesel 2019). However, overactive performance monitoring in OCD does not lead to an increase in decision‐making performance (Gruner and Pittenger 2017; Riesel et al. 2019). The transition between action strategies can only be achieved by suppressing habitual responses following error detection (Gillan et al. 2011; Amaya and Smith 2018). Therefore, our findings suggest that the CI group may be characterized by less habitual behavior. This hypothesis needs to be tested with further experimental studies in cognitive subtypes. In support of this hypothesis, the severity of compulsive symptoms, which might be associated with habit dysregulation and excessive habit formation, was lower in the CI subgroup. A recent symptom‐based profiling study also found that the severity of compulsive symptoms was associated with cognitive impairment (Wu et al. 2022). Consistent with our findings, a community‐based study that screened for OCD symptoms reported significant global cognitive impairments, including memory, inhibitory control, and working memory in the group characterized by repetitive actions and compulsions (Kennedy et al. 2023). In the only study to categorize OCD patients into neurocognitive subtypes, no differences were found between the CI and GI performance groups in terms of symptom severity, duration of illness, age, gender, education level, and employment status (Malcolm et al. 2021). However, this preliminary study included a diagnostically heterogeneous population, and comparisons within cognitive subgroups were not made specifically for OCD.

There were no differences in symptom frequencies between cognitive clusters. Our study is the first to investigate differences in symptom profiles between cognitive clusters in OCD. To date, differences in symptom dimensions in terms of neuropsychological functions have been investigated using a symptom‐oriented approach, revealing partial overlaps and differences in neuropsychological functions among obsession types (Lawrence et al. 2006; van den Heuvel et al. 2009; Omori et al. 2012; Abramovitch et al. 2015). These differences in neuropsychological performance among symptom dimensions have been suggested to indicate distinct neural sources. However, neuroimaging studies investigating the neural correlates underlying symptom dimensions have provided inconsistent results (Mataix‐Cols et al. 2004; Gilbert et al. 2008; van den Heuvel et al. 2009; Harrison et al. 2013). Variability of symptoms over time and the co‐occurrence of multiple obsessions/compulsions may be the cause of inconsistent results. Therefore, the contribution of symptom‐level classifications may have limited evidential value, and investigating differences between symptoms in patients classified according to neurobiological parameters might be more useful for elucidating etiopathogenesis (Cuthbert 2015; Vaghi 2021; Ding et al. 2024). The interaction of a relatively small number of biotypes with environmental factors, shaped by common neurobiological mechanisms underlying different symptom clusters, may account for symptom diversity (Wang et al. 2023). Further studies investigating symptom profiles in neurocognitive subtypes, as well as other clinical features and environmental influences, may increase our understanding of the relationship between neurobiological mechanisms and clinical presentation in OCD.

Consistent with our hypothesis, years of education were significantly lower in the GI group. Cognitive ability is known to be associated with low academic achievement and years of education completed (Shi and Qu 2022). Several studies show that cognitive subgroups in schizophrenia and bipolar disorder may reflect neurobiological differences that determine academic performance (Bora et al. 2016; Carruthers et al. 2022; Bora et al. 2023). Neurodevelopmental abnormalities can be associated with both poor cognitive reserve (and lower educational attainment) and future risk of OCD. Similar to findings in schizophrenia and bipolar disorder (Bora et al. 2023), one might expect to see increased problems in social and motor development as antecedents of OCD with pronounced cognitive impairment. In addition, low educational attainment may also be related to the interaction of developmental and environmental factors such as income level and parental education level. These variables were not assessed in our study. Further studies using the cognitive clustering method in OCD may provide a more holistic approach by considering environmental and developmental variables.

AoO was significantly higher in the GI group. In support of our findings, increased AoO is associated with greater impairment in set‐shifting, processing speed, and verbal fluency (Roth et al. 2005; Hwang et al. 2007; Kim et al. 2020). At the same time, both cognitive clusters showed similar patterns, and there was a significant negative correlation between AoO and processing speed. However, the limited number of studies investigating the relationship between AoO and neurocognitive performance in OCD and the use of different age thresholds in studies with a categorical approach makes it difficult to interpret the results (Abramovitch et al. 2015; Geller et al. 2021). Moreover, a categorical approach to AoO runs the risk of assigning significance to nonsignificant differences. Indeed, in our study, AoO is distributed over a relatively narrow range, and categorization at any given point may lead to artificial results. In our sample, the mean AoO of the two cognitive subgroups is above the various thresholds used in the literature. Nevertheless, our results are consistent with studies in the literature showing that later AoO is associated with worse neurocognitive functioning. In addition, it is important to assess the effect of disease duration (Nakao et al. 2009). In our study, the disease duration was relatively short, and there was no significant difference between the groups. Follow‐up studies may be useful to clarify the relationship between AoO and neurocognitive functions in cognitive subtypes and to determine whether cognitive impairment progresses or remains stable over time.

This study has several limitations. First, due to its cross‐sectional design, the stability of cognitive subgroups over the course of the illness and the change in their relationship with clinical variables could not be assessed. Second, the neuropsychological variables did not include tests that have been shown to be impaired in OCD, such as visual memory, reward learning, and decision making. Third, the sample size was not large enough to compare primary obsessions across cognitive clusters. At the same time, in our study, the lack of evaluation of the relationship between the severity of individual symptom dimensions and neurocognitive performances is another limitation. In future studies, comparing symptom severity across cognitive subgroups and assessing their relationships with cognitive performance could be an important approach in understanding the etiopathogenesis. Fourth, the inter‐rater variability of neuropsychological assessments conducted at different centers has not been evaluated. Fifth, confounding variables such as family history, baseline IQ, dose of psychotropics, duration of treatment, treatment resistence, and severity of comorbid anxiety symptoms were not assessed. Studies that evaluate these variables in cognitive subgroups in OCD could lead to clearer conclusions and help us gain insights into genetic predispositions within cognitive clusters. Finally, the presence of MDD was used as an exclusion criterion to control for its confounding effect on cognitive performance and to more clearly assess the cognitive heterogeneity in OCD. On the other hand, the exclusion of MDD may reduce the generalizability of the results.

In conclusion, our findings indicate that poorer cognitive functioning represents a subgroup with higher compulsion severity. This result may provide insights for future research into understanding the reasons behind failures in suppressing compulsions and therapeutic interventions. Better characterization of cognitive heterogeneity in OCD could potentially facilitate genetic and biological studies, as well as help clinicians and researchers develop more effective intervention strategies targeting cognitive deficits. Follow‐up neuroimaging studies will also provide an opportunity to assess the temporal stability of subgroups and their neural correlates.

## Author Contributions

**Emre Mısır**: writing–original draft, formal analysis, data curation, investigation, resources. **Raşit Tükel**: writing–review and editing, resources. **Berna Binnur Akdede**: writing–review and editing, resources. **Emre Bora**: conceptualization, writing–review and editing, formal analysis, methodology, investigation.

## Ethics Statement

This study was approved by the Baskent University Institutional Review Board and Ethics Committee (Project no and date: KA24/310/18.09.2024).

## Consent

Written informed consent was obtained from the participants during data collection for each study constituting the database of this study.

## Conflicts of Interest

The authors declare no conflicts of interest.

### Peer Review

The peer review history for this article is available at https://publons.com/publon/10.1002/brb3.70375.

## Supporting information



Supplementary Materials.

## Data Availability

The data that support the findings of this study are available from the corresponding author upon reasonable request. The data are not publicly available due to privacy or ethical restrictions.
