# Supplementary material for: Clinical Characteristics of Cognitive Subgroups of Obsessive Compulsive Disorder
Source: Brain Behav. 2025 Mar 13;15(3):e70375. doi: 10.1002/brb3.70375 (PMC11904946; doi:10.1002/brb3.70375)
Supplement: Supplementary file 1 — Supplementary Materials. [file BRB3-15-e70375-s001.docx]

**Table S1.** Demographic Characteristics in Patients and Healthy Controls

|  | OCD  (n=135) | HC  (n=106) | Statistics |
| --- | --- | --- | --- |
| Age | 29.5 (9.5) | 29.9 (9.5) | t(239)=-0.3  p=0.76 |
| Gender (F/M) | 89/46 | 56/50 | χ2 (1)= 4.2  ***p=0.039^*^*** |
| Married/No Married | 50/85 | 50/56 | χ2 (1)=2.51  p=0.11 |
| Employed/Unemployed | 46/89 | 51/55 | χ2 (1)=4.86  ***p=0.027^*^*** |
| Education | 11.1 (4) | 12.5 (4.4) | t(239)=--1.4  ***p***=***0.01^*^*** |
| HAMD-17 | 6.5 (4) | 1.6 (2) | t(239)=-11.8  ***p<0.001^***^*** |

OCD: Obsessive compulsive disorder, HC: Healthy controls

***p<0.05, **p<0.01, ***p<0.001**

**Table S2**. Neurocognitive Performances in Patients and Healthy Controls

|  | OCD  (n=135) | HC  (n=106) | Statistics |
| --- | --- | --- | --- |
| *Verbal memory* | -0.3 (0.8) | 0 (1) | t(239)=-*2.4*  ***p=0.018**** |
| CVLT Trial 1 | 6.8 (2.1) | 7.5 (2.4) | t(239)=-*2.4*  *p=****0.016**** |
| CVLT Learn | 52.1 (10.2) | 56.1 (11.3) | t(239)=-*2.8*  ***p=0.005^**^*** |
| CVLT Del | 11.7 (2.8) | 12.2 (2.9) | t(239)=-*1.2*  p=0.25 |
| *Executive functions* | 0.1 (0.5) | 0 (1) | t(239)=-*3.2*  ***p=0.002***** |
| WCST Per | 18.5 (12.5) | 15.1 (10.5) | t(239)=-*2.2*  ***p=0.02**** |
| WCST Cat | 4.4 (1.9) | 4.8 (1.6) | t(239)=-*1.6*  *p=0.1* |
| TMT-B Dur | 110.3 (71.8) | 79.8 (48.9) | t(239)=-*3.7*  ***p<0.001^**^*** |
| *Verbal fluency* | -0.3 (0.9) | 0 (1) | t(239)=--4.8  ***p<0.001*** |
| COWAT | 33.9(14.1) | 43.0 (15.4) | t(239)=*-4.8*  ***p<0.001*** |
| *Processing speed* | -0.3 (0.9) | 0 (1) | t(239)=-3.9  p=***0.001^**^*** |
| TMT-A Dur | 41.2 (18.4) | 32.3 (16.4) | t(239)=*-3.9*  ***p<0.001^***^*** |
| *Working memory* | 0.0 (0.6) | 0 (1) | t(106)=-*1.9*  ***p=0.05**** |
| ACTT total | 48.2 (8.9) | 49.9 (8.3) | t(239)=*-1.1*  p=0.29 |
| DS- F | 6.1 (2.1) | 6.9 (2.6) | t(239)=*-1.8*  p=0.07 |
| DS- B | 6 (2.2) | 7.2 (3.0) | t(239)=-*2.4*  ***p=0.019**** |

CVLT: California Verbal Learning Test, CVLT Learn: CVLT Learning (Trial 1 to 5), CVLT Del: CVLT Delayed recall (Trial 7), WCST: Wisconsin Cart Sorting Test, WCST Per: WCST perseverative errors, WCST Cat: WCST completed category, TMT-B Dur: Trail Making Test duration to complete, COWAT: Controlled Oral Word Association test, ACTT: Auditory Consonant Trigram Test, DS-F: Digit span forwards test, DS-B: DS backwards test

***p<0.05, **p<0.01, ***p<0.001**


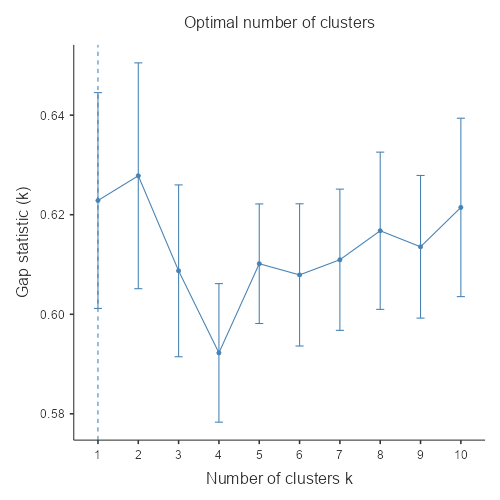


**Figure S1.** Optimal number of clusters for patients with OCD


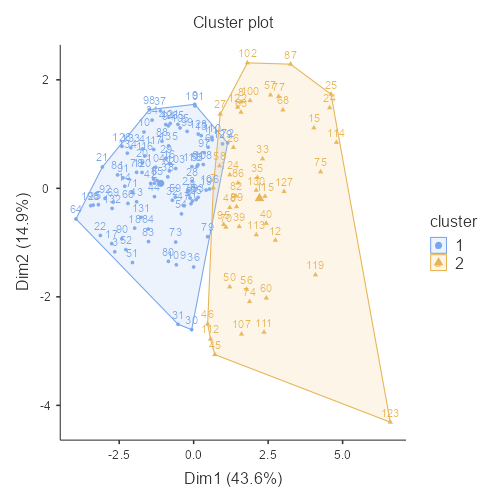


**Figure S2**. The cluster plot of patients with OCD
